# The Integrated Analysis of miRNome and Degradome Sequencing Reveals the Regulatory Mechanisms of Seed Development and Oil Biosynthesis in Pecan (*Carya illinoinensis*)

**DOI:** 10.3390/foods13182934

**Published:** 2024-09-16

**Authors:** Kaikai Zhu, Lu Wei, Wenjuan Ma, Juan Zhao, Mengyun Chen, Guo Wei, Hui Liu, Pengpeng Tan, Fangren Peng

**Affiliations:** 1 Co-Innovation Center for Sustainable Forestry in Southern China, Nanjing Forestry University, Nanjing 210037, China; kkzhu@njfu.edu.cn (K.Z.); wl18236975076@163.com (L.W.); mabuer1998@163.com (W.M.); cxyl@njfu.edu.cn (J.Z.); hehecmy@163.com (M.C.); tanpengpeng2002@163.com (P.T.); 2 College of Horticulture and Landscape Architecture, Yangzhou University, Yangzhou 225009, China; gwei@yzu.edu.cn; 3 State Key Laboratory of Crop Genetics & Germplasm Enhancement and Utilization, Ministry of Agriculture and Rural Affairs Key Laboratory of Biology and Germplasm Enhancement of Horticultural Crops in East China, College of Horticulture, Nanjing Agricultural University, Nanjing 210095, China; liuhui@njau.edu.cn

**Keywords:** *Carya illinoinensis*, expression patterns, microRNA, oil biosynthesis, seed development

## Abstract

Pecan seed oil is a valuable source of essential fatty acids and various bioactive compounds; however, the functions of microRNAs and their targets in oil biosynthesis during seed development are still unknown. Here, we found that the oil content increased rapidly in the three early stages in three cultivars, and that oleic acid was the predominant fatty acid component in the mature pecan embryos. We identified, analyzed, and validated the expression levels of miRNAs related to seed development and oil biosynthesis, as well as their potential target genes, using small RNA sequencing data from three stages (120, 135, and 150 days after flowering). During the seed development process, 365 known and 321 novel miRNAs were discovered. In total, 91 known and 181 novel miRNAs were found to be differentially expressed, and 633 target genes were further investigated. The expression trend analysis revealed that the 91 known miRNAs were classified into eight groups, approximately two-thirds of which were up-regulated, whereas most novel miRNAs were down-regulated. The qRT–PCR and degradome sequencing data were used to identify five miRNA- target pairs. Overall, our study provides valuable insights into the molecular regulation of oil biosynthesis in pecan seeds.

## 1. Introduction

Pecan [*Carya illinoinensis* (Wangenh.) K. Koch], a commercially valuable nut tree species, which is native to North America, has been widely cultivated [1]. Pecan nuts are consumed as snacks, which are valuable sources of unsaturated fatty acids (FAs), flavonoids, tocopherols, vitamins, starch, and phenolic compounds, and have become one of the most popular edible nuts in the global nut industry [2]. The pecan nut is not only a culinary delight, but it is also a valuable source of oil; notably, mature pecan embryos contain more than 70% oil (70–79%) [3]. Pecan oil is rich in polyunsaturated and monounsaturated FAs, but it contains a low amount of saturated FAs, which have a positive influence on health benefits [4,5]. In addition, pecan oil has a pleasant, nutty taste and is ideal for sweet dishes and salads. Thus, exploring the molecular mechanisms of pecan seed development and the oil biosynthesis process will lay the foundation for enhancing nut quality and breeding high-yield oil-type pecan cultivars.

The biosynthesis of oil in plants is a complex, multistep process regulated by intricate networks, and knowledge of plant oil accumulation is based on the studies of oil seeds. The first step in the biosynthesis of oil in plants is the biosynthesis of FAs in plastids, primarily catalyzed by FA synthase. Triacylglycerols (TAGs), which are the major form of stored lipids, are synthesized in the endoplasmic reticulum [6]. Finally, TAGs combine with oleosin to form oil bodies in the cytoplasm [7]. FAs have multiple functions in plants, such as serving as signaling molecules and membrane components, and playing roles in plant defense. The process of pecan seed development lasts more than four months, typically from May to October, and FAs significantly accumulate in the later stages [3]. 

Given the increasing interest in plant oil quality and quantity, various approaches, including conventional breeding, transcriptomics, metabolomics, proteomics, and quantitative trait loci (QTL) mapping have been employed. MicroRNAs (miRNAs) are a type of endogenous non-coding small RNA of approximately 22 nucleotides in length. Numerous miRNAs have been found to play key roles in a wide range of biological processes, including growth, development, and stress response [8]. They can decrease gene expression by guiding target mRNA degradation or inhibiting translation post-transcriptionally [9]. In plants, miRNAs play pivotal roles in the process of the seed development process [10,11]. For example, OsmiR396 and OsmiR408 could substantially increase the seed size in rice [12]. For oil crops, high-throughput sequencing has been used to identify key miRNAs during seed development, including soybean, walnut, etc. In soybean, 31 previously validated miRNAs, including 14 isoforms of miRNAs and 17 novel miRNAs, have been found to be involved in seed development [13]. Sixteen miRNA-mRNA signaling modules are identified to participate in oil accumulation and the FA synthesis processes in walnuts, and four key miRNAs were closely related to oil accumulation [14]. Previous studies have demonstrated that oil accumulates during pecan embryo development and identified key genes associated with lipid biosynthesis using high-throughput RNA-Seq technology [3,7,15]. 

Several studies have reported the involvement of miRNAs in pecan graft union formation, revealing their potential roles [16,17]. However, limited information about the miRNAs involved in pecan seed development or oil accumulation is available. In this study, the fatty acid content and composition of developing pecan seeds were analyzed, and sequencing data were generated on the miRNAome and degradome in pecan embryos collected at different developmental stages. Our findings may aid in elucidating the key regulatory networks involved in oil biosynthesis during pecan seed development. This study aimed to increase our understanding of the regulatory mechanisms underlying oil biosynthesis in pecan seeds. The expression data of miRNAs and potential candidate miRNA-mRNA regulatory networks can be applied to improve the quality of pecan nuts and increase the oil yield.

## 2. Materials and Methods

### 2.1. Plant Materials and Sample Collection

The pecan trees were cultivated in a pecan orchard situated in Jurong city, China (31°52′47″ N, 119°9′5″ E). The orchard experienced a mean annual precipitation of 1018.6 mm and an annual mean temperature of 15.6 °C. The pecan fruits were meticulously harvested at various developmental stages, including 120 (DAF120), 135 (DAF135), 150 (DAF150), 165 (DAF165), and 180 (DAF180) days after flowering from healthy nine-year-old trees from August to October. Following the removal of the pericarp and seed coat, the embryos were rapidly frozen in liquid nitrogen and stored at −80 °C for future experiments. Each sample was harvested from at least ten pecan fruits, with three biological repetitions employed for each timepoint.

### 2.2. An Analysis of the Oil Content and Components and the Soluble Sugar Content 

The embryo samples from three pecan cultivars (‘Pawnee’, ‘Jinhua’, and ‘Mahan’) that were harvested at five different stages were oven-dried at 60 °C until they reached a constant weight. The oil content of the pecan embryos was determined using the Soxhlet extraction method [18]. The samples were crushed, and then placed in a Soxhlet extraction apparatus using petroleum ether as a solvent. Oil content analyses were performed with three biological replicates.

The fatty acid components were measured via gas chromatography–mass spectrometry (Agilent GC–MS 7890A, Santa Clara, CA, USA) according to previously described methods [19]. According to the GC–MS results, the percentage of each fatty acid component was calculated via the peak area normalization method. 

For the total soluble sugar content assay, 0.2 g embryo samples were collected and heated in a boiling water bath for 1 h, and the resulting extract was naturally cooled. The soluble sugar content was subsequently measured by a plant soluble sugar content detection kit (Keming Biotechnology Co., Ltd., Suzhou, China) following the manufacturer’s instructions. The absorbance of the soluble sugar content was measured at 620 nm.

### 2.3. Small RNA Library Construction and Sequencing

For small RNA sequencing, embryo samples from three stages (DAF120, DAF135, and DAF150) that were collected from the genome-sequenced cultivar ‘Pawnee’ were employed. The total RNA was extracted via a TRIzol kit (Invitrogen, Carlsbad, CA, USA) following the manufacturer’s protocol, and RNA molecules ranging from 18 to 30 nucleotides (nt) in size were enriched by polyacrylamide gel electrophoresis (PAGE). Nine small RNA libraries from the three different stages were constructed, and miRNA sequencing was conducted using the Illumina HiSeq Xten platform by Genedenovo Biotechnology Co., Ltd. (Guangzhou, China). 

### 2.4. Analysis of Small RNA Sequences

The clean reads were used for the miRNA analysis after the removal of undesirable reads, such as low-quality reads, poly-A-containing reads, sequence adapter-containing reads, etc. The clean reads were further aligned with small RNAs in the GeneBank and the Rfam database to eliminate tRNAs, scRNAs, rRNAs, snoRNAs, and snRNAs. The remaining clean reads were subsequently compared against the miRbase 22 database to identify known miRNAs. These unique sequences, which could be compared with the reference genome but could not be compared with the pre-miRNAs in miRbase, were considered as novel candidate miRNAs [20].

### 2.5. RNA Isolation and Transcriptome Sequencing

The total RNA was isolated using the TRIzol kit, and genomic DNA contamination was eliminated using RNase-free DNase I (Qiagen, Hilden, Germany). The RNA quality and concentration were assessed via agarose gel electrophoresis and the NanoDrop 2000 (Thermo Fisher Scientific, Wilmington, NC, USA). For each sample, 1 μg of RNA was reverse transcribed to cDNA, and nine cDNA libraries were constructed and sequenced with an Illumina NovaSeq 6000 platform (Genedenovo, Guangzhou, China). 

### 2.6. Differentially Expressed miRNAs and Target Prediction 

The miRNA counts in each library were further calculated and normalized to transcripts per million (TPM) values. To identify the differentially expressed miRNAs across the groups, the edgeR package was used [21]. A miRNA was deemed significantly differentially expressed if it exhibited a fold change ≥ 2 (*p* < 0.05). The expression trends of the significantly differentially expressed miRNAs (DEMs) were classified into groups using Short Time-series Expression Miner (STEM), software version 1.3.13, with the default parameters [22].

The target genes of the miRNAs were further screened from the transcriptome data, and patmatch v1.1 software (https://www.arabidopsis.org/cgi-bin/patmatch/nph-patmatch.pl (accessed on 24 November 2023)) was used to predict miRNA targets from the differentially expressed genes. 

A degradome analysis was further conducted to validate the miRNA targets. The total RNA from the nine embryo samples of the developing pecan seeds was pooled to create the degradome library. The degradome library was subsequently sequenced using an Illumina HiSeq 2000 system by Genedenovo Biotechnology Co., Ltd. (Guangzhou, China). After low-quality reads, contaminants and adaptors were removed via a Perl script and the clean reads were aligned with the RNA-Seq data of the pecan samples. The plant small RNA target prediction server psRNA Target (https://www.zhaolab.org/psRNATarget/ (accessed on 24 November 2023)) was applied to assess the complementarity between candidate miRNAs and the target site of genes using the default parameters [23].

The Kyoto Encyclopedia of Genes and Genomes (KEGG) is a pathway-related publicly available database (https://www.genome.jp/kegg/ (accessed on 24 November 2023)), and KEGG pathway analyses were used to investigate the roles of the putative miRNA target genes in biological functions. 

### 2.7. Validation of the Expression Patterns of miRNAs and Target Genes 

A quantitative real-time reverse-transcriptase PCR (qRT–PCR) was applied to quantify the expression patterns of the miRNAs and their target genes during pecan seed development. The reverse-transcription reactions were performed using the miRNA 1st Strand cDNA Synthesis Kit (Vazyme Biotech, Nanjing, China) to obtain cDNAs. A qRT–PCR analysis was carried out with miRNA Universal SYBR qPCR Master Mix (Vazyme Biotech, Nanjing, China) on an ABI 7500 Real-Time PCR system (Applied Biosystems, Foster City, CA, USA). U6 small RNA served as the internal control for the normalization of miRNA expression [17]. 

Verification of the expression of the target genes was also performed via a qRT–PCR analysis with the TB Green Premix Ex Taq II Kit (Takara, Beijing, China). A housekeeping actin gene (*CiPaw.03G124400*) was selected as the internal reference for normalization [2]. All primers were designed via the IDT Primer Quest tool (https://sg.idtdna.com/PrimerQuest/Home/Index (accessed on 24 November 2023)) and listed in Table S1. The relative expression of the miRNAs and target genes compared with that of the controls was calculated using the 2^−∆∆Ct^ method [24]. 

### 2.8. Statistical Analysis

The expression data for the miRNAs and their targets from at least three independent biological replicates during pecan seed development were presented as the mean values and standard error (SE). Significant differences at different timepoints were analyzed by Duncan’s multiple range test (*p* < 0.05) with IBM SPSS Statistics v25 (SPSS Inc., Chicago, IL, USA). A correlation analysis was performed via the R language for two parallel experiments. 

## 3. Results

### 3.1. Oil and Sugar Contents and Fatty Acid Composition Analyses

**Figure 1:**
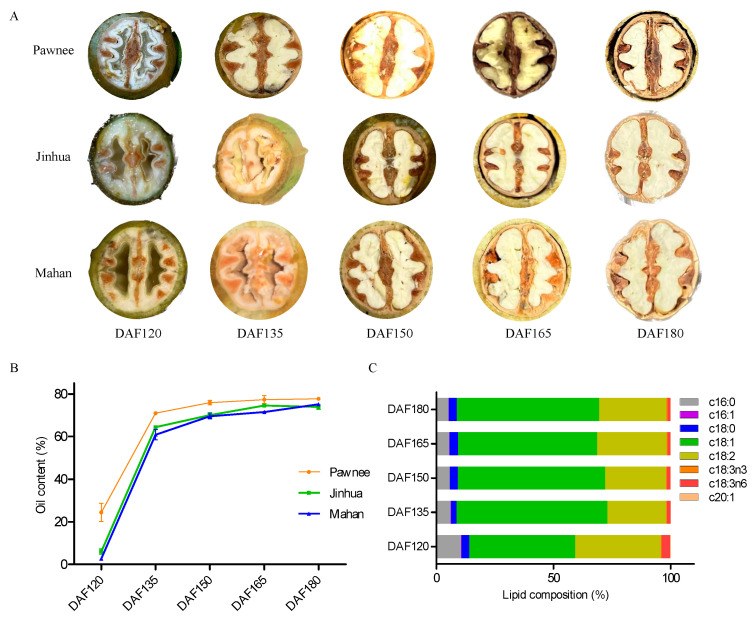
Oil content and fatty acid composition in developing pecan embryos. (**A**) Observation of morphological characteristics during five stages of pecan seed development. Bar = 1 cm. (**B**) Oil contents of seed samples at five stages from three pecan cultivars. (**C**) Changes in fatty acid composition at different stages in pecan embryos. c16:0, palmitic acid; c16:1, palmitoleic acid; c18:0, stearic acid; c18:1, oleic acid; c18:2, linoleic acid; c18:3n3, α-Linolenic acid; c18:3n6, γ-Linolenic acid; and c20:1, Eicosenoic acid.

In this study, embryo samples at five developmental stages from three pecan cultivars (‘Pawnee’, ‘Mahan’, and ‘Jinhua’) were collected. We observed that the pecan embryos changed from the watery stage (DAF120) to the creamy yellow and full stage (DAF180) (Figure 1A). The oil content of the pecan seed samples was analyzed, and we found that the seeds from the ‘Pawnee’ cultivar consistently presented a higher oil content than did those from the other two cultivars across different developmental stages (Figure 1B). The oil content rapidly increased from DAF120 to DAF135 in all three cultivars. At the early cotyledon stage (DAF120), the oil content was 24.43% in ‘Pawnee’; however, ‘Jinhua’ and ‘Mahan’ showed lower percentages, at 6.05% and 2.74%, respectively. During the DAF135 stage, the embryos developed a flavescent surface surrounding the fleshy inside. The oil content increased to 71.01% in ‘Pawnee’, and 64.05% and 60.87% in ‘Jinhua’ and ‘Mahan’, respectively. At the fully mature stage (DAF180), the oil content exceeded 70% in all of the pecan samples. These results suggested that oil accumulation in pecan embryos is a major feature from DAF120 to DAF135.

Acetyl-CoA is the important precursor for the biosynthesis of lipids, and sugars are its primary source [25]. In the pecan embryo samples, we observed a gradual decline in the soluble sugar content from DAF120 to DAF150, whereas the oil content gradually increased, suggesting a potential link between the sugar and oil content in the pecan seeds (Figure S1).

The FA compositions of pecan oil were also evaluated at five stages of seed development (Figure 1C). Eight FAs were detected in the five stages, including two types of saturated FAs and six types of unsaturated FAs. Stearic acid (c18:0) and palmitic acid (c16:0) were two of the main components of saturated fatty acids, and they were maintained at low levels (<15%) across all five stages. In contrast, the levels of unsaturated fatty acids were relatively high, with oleic acid (c18:1) and linoleic acid (c18:2) being the top two unsaturated FA components in pecan embryos, accounting for more than 80% at all stages. The oleic acid content quickly increased from 45% (DAF120) to 64% (DAF135), whereas the linoleic acid content decreased from 35% to 28%. 

### 3.2. Sequence Analysis of Small RNAs

**Figure 2:**
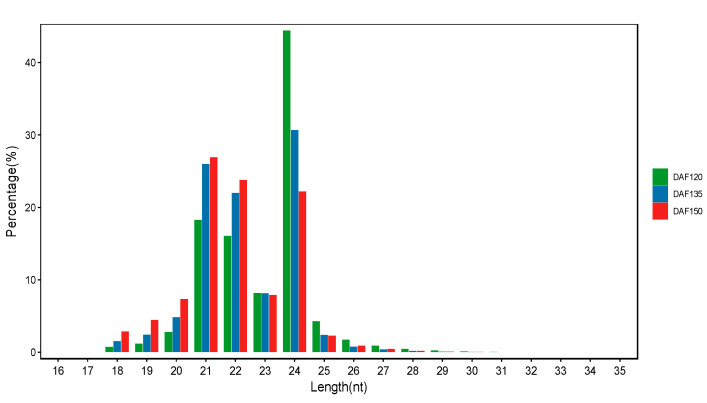
Length distribution of small RNA from the seeds of the ‘Pawnee’ cultivar of pecan at three developmental stages.

A rapid increase in oil content was detected from DAF120 to DAF150 during pecan seed development (Figure 1B). To identify miRNAs associated with the development and oil accumulation of pecan seeds, nine small RNA libraries were constructed and sequenced using the pecan embryo samples collected at 120, 135, and 150 days after flowering. The Pearson correlation coefficient between the pecan embryo samples was calculated and analyzed to evaluate the consistency of the different biological replicates (Figure S2). We found that every two replicates had a high correlation coefficient, indicating that the results were reliable and operational stable. The high-throughput sequencing results revealed that 10,266,300 to 12,529,371 clean reads were obtained from the libraries after low-quality reads were removed (Table S2). After investigating the size distribution of the unique small RNAs in the nine libraries (Figure 2), we found that the length of the sequenced small RNA reads ranged from 18 to 35 nt, with the majority falling between 21 and 24 nt. For the DAF120 and DAF135 stages, the most abundant sRNAs were 24 nt sRNAs [16]. However, for the DAF150 stage, 21 nt sRNAs were the most abundant in our libraries, which is consistent with previous results [17].

### 3.3. Identification of Known and Novel MicroRNAs in Developing Pecan Seeds

**Figure 3:**
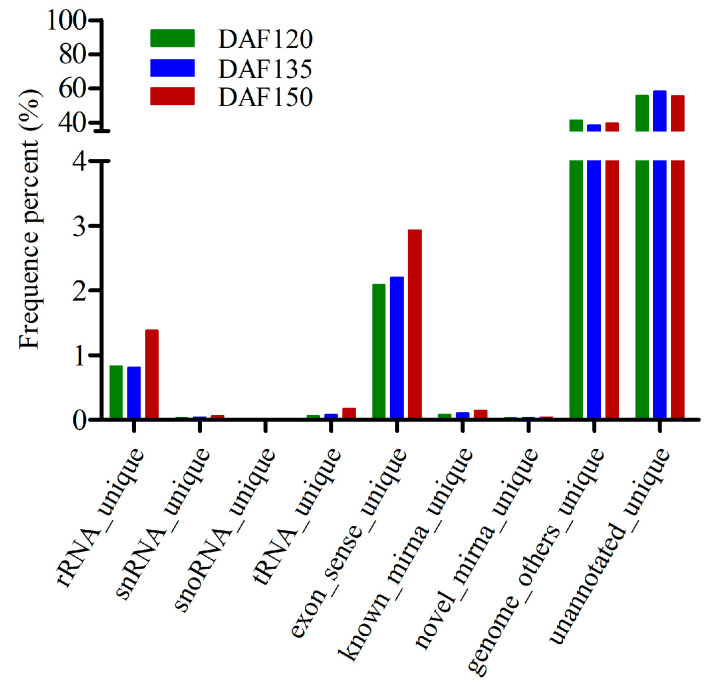
Composition of the small RNA in nine libraries.

By alignment with the GeneBank and Rfam databases, the clean reads were classified into different small RNA types, and no significant difference in the percentage of small RNA types was detected (Figure 3). A total of 365 known miRNAs were identified in the nine libraries (Table S3), and they were classified into 296 families. For the DAF120 samples, only 152 known miRNAs were identified, while the number of known miRNAs increased to 189 and 185 in the DAF135 and DAF150 samples, respectively (Table 1). The expression patterns of the 365 known miRNAs, which exhibited substantial differences in expression levels, were investigated. The TPM values of 70.14% (256/365) of the known miRNAs were less than five in all three stages, indicating that these miRNAs were at low levels (Figure S3A). However, several miRNAs, including miR166-y, miR167-x, miR482-y, and miR396-x, presented high expression levels. 

In total, 321 novel miRNAs were found from the nine libraries (Table S3), and all of the miRNA precursors contained typical stem-loop structures. The novel miRNAs varied in length from 18 to 25 nt, with 24 nt being the most abundant (Table S4). Interestingly, the expression profiles of the novel miRNAs revealed that only three of them were expressed at low levels (Figure S3B). 

Subsequently, the first nucleotide bases of the miRNAs were analyzed. We found that the most abundant base for the first nucleotide was uracil (U, 40.78%), followed by adenosine (A, 34.29%) and cytosine (C, 20.74%) (Figure S4).

### 3.4. Differentially Expressed miRNAs during Pecan Seed Development

The differentially expressed miRNAs were identified via a bioinformatics analysis. A total of 272 miRNAs were identified as DEMs, comprising 91 known miRNAs and 181 novel miRNAs. A total of 133 and 149 DEMs were identified from the two groups of DAF120 vs. DAF135 and DAF135 vs. DAF150, respectively (Table S5). However, the number of DEMs increased to 204 in the DAF120 vs. DAF150 comparison, indicating that more DEMs were found in the later stages of seed development (Table S5). 

**Figure 4:**
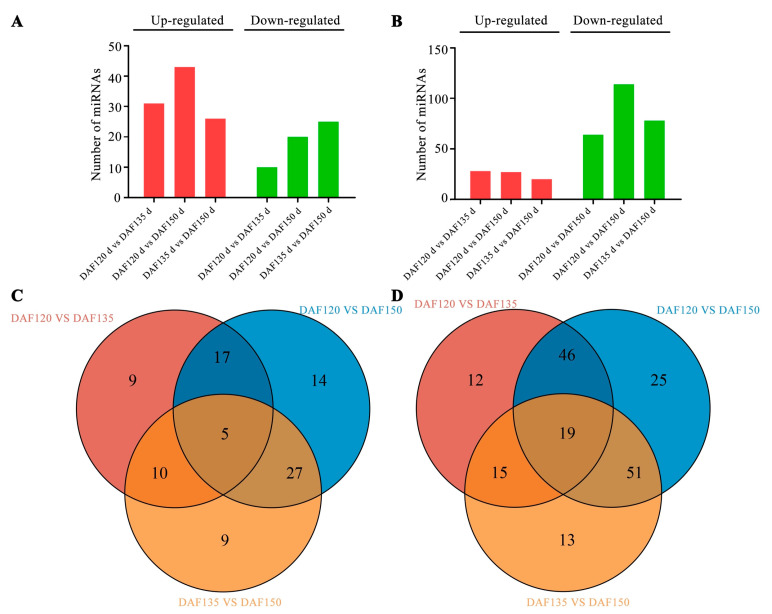
Differentially expressed miRNAs in three developmental stages of pecan seeds. (**A**) The number of the known DEMs between the different groups. (**B**) The number of novel DEMs between the different groups. (**C**) Venn diagrams of the known DEMs. (**D**) Venn diagrams of the novel DEMs.

In the DAF120 vs. DAF135 comparison, a total of 41 known miRNAs were identified, 31 of which were up-regulated and 10 of which were down-regulated (Figure 4A). In total, 63 known DEMs were identified in the DAF120 vs. DAF150 comparison, 43 of which were up-regulated and 20 of which were down-regulated. A total of 51 known DEMs were found in the DAF135 vs. DAF150 comparison, of which 26 were up-regulated and 25 were down-regulated. Surprisingly, we found that the number of up-regulated known DEMs was consistently greater than the number of down-regulated DEMs in each group (Figure 4A). However, for the novel DEMs, the majority were down-regulated (Figure 4B).

Moreover, the number of common and unique DEMs across various groups was visually shown in a Venn diagram. According to the Venn diagram analysis, five known DEMs (miR2950-x, miR167-y, miR157-x, miR5225-x, and miR7122-y) were identified in all three comparison groups (Figure 4C), and nineteen novel DEMs were also found in all three groups (Figure 4D). 

### 3.5. Expression Trends of DEMs during Seed Development and Oil Biosynthesis in Pecan 

**Figure 5:**
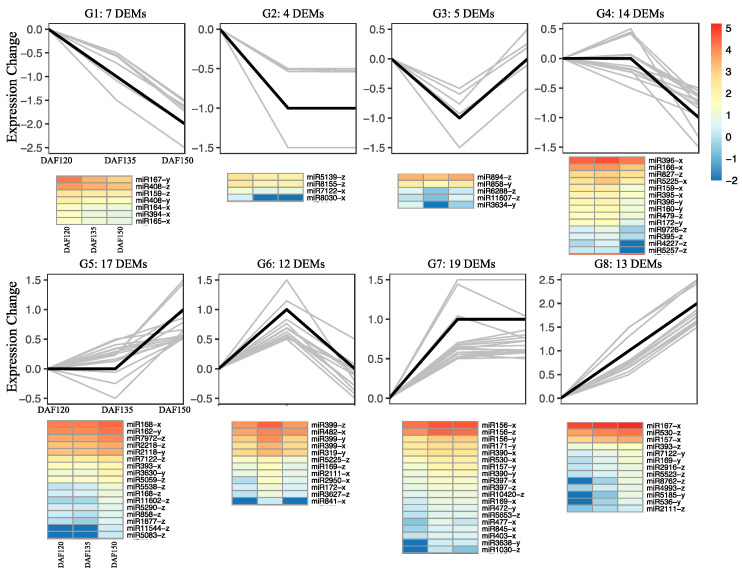
Expression trends of known DEMs in three developmental stages of pecan seeds. The number of DEMs in each group is listed at the top of each group.

To study the expression patterns of the differentially expressed miRNAs during pecan seed development, we further investigated the expression trends of the DEMs on the basis of their TPM values. In total, the 91 known DEMs were classified into eight groups according to clustering analysis, ranging from 4 to 19. Each group was hierarchically clustered and presented as heatmaps (Figure 5). Thirteen DEMs in group 8 (G8) gradually increased in expression during seed development, whereas seven DEMs in G1 gradually decreased in expression. Notably, the largest group (G7), with 17 DEMs, presented a significant increase in expression from DAF120 to DAF135. However, the second largest group (G5), with 17 DEMs, increased significantly from DAF135 to DAF150. 

The expression patterns of the novel DEMs were also investigated (Figure S5), and the clustering results revealed that the most novel DEMs were down-regulated during pecan seed development.

### 3.6. Identification and Characterization of miRNA Targets

In general, miRNAs and their target genes are negatively regulated. According to the prediction results, out of the 686 identified differentially expressed miRNAs, only 76 miRNAs had 633 predicted targets (Table S6). Among the 365 known miRNAs, 60 miRNAs had predicted target genes; however, for the 321 novel miRNAs, only 16 miRNAs had predicted target genes. Some known miRNA families, such as miR156, miR159, and miR168, targeted different potential genes, suggesting that these members of the same family might be involved in regulating multiple genes in pecan seeds. 

**Figure 6:**
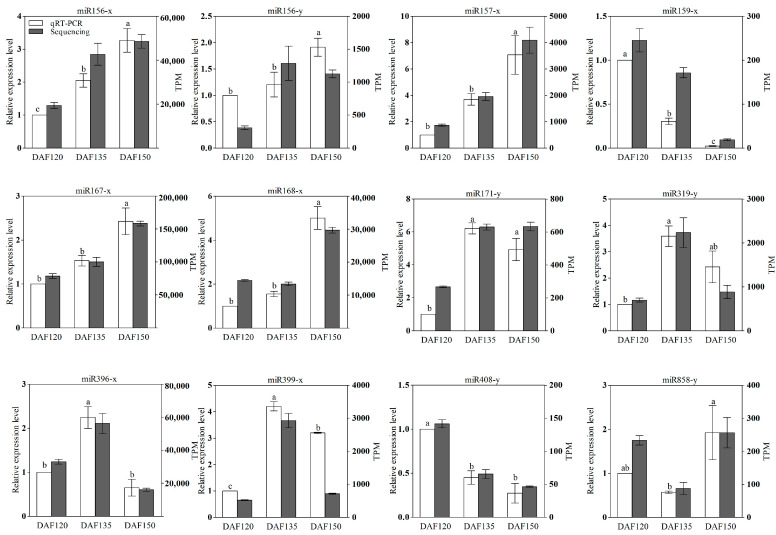
qRT–PCR validation of miRNAs during three stages of seed development in pecan seeds. Values are means ± SE of three replicates, and bars with different letters were significantly different at *p* < 0.05 using Duncan’s multiple range test.

To validate the accuracy of the miRNA sequencing results, we selected 12 DEMs from the 91 known DEMs (Figure 5) as candidates, and we applied a qRT–PCR assay to analyze their expression levels during seed development at three timepoints (120, 135, and 150 days after flowering). The expression patterns of all 12 miRNAs were consistent with the high-throughput results (Figure 6). Compared with those of DAF120, the expression of ten of these genes was up-regulated, and we found that miR171y, miR319y, miR396x, and miR399x presented the highest expression levels at DAF135. Only two DEMs (miR159x and miR408y) were down-regulated during pecan seed development. 

**Figure 7:**
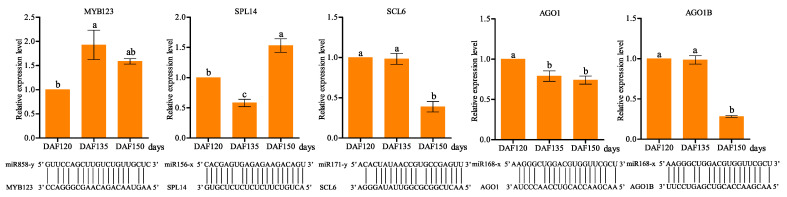
Validation of the target genes during three stages of seed development in pecan, showing the expression patterns of five candidate target genes and a prediction of the binding sites of miRNAs in targets using the psRNA Target. Values are means ± SE of three replicates, and bars with different letters were significantly different at *p* < 0.05 using Duncan’s multiple range test.

Additionally, the expression patterns of the five target genes of the DEMs were further investigated using a qRT–PCR. The expression profiles of the four miRNAs were compared with those of the target genes, and the target genes with putative target sites of the DEMs were detected via the microRNA target matching analysis (Figure 7), revealing an inverse correlation between the expression of the miRNAs and their targets. 

For a comprehensive understanding of the potential functions of these target genes in pecan seed development, KEGG annotations were investigated for the top 20 pathways based on the basis of the q value (Figure S6). Among the enrichment results of the DAF120 vs. DAF135 and DAF120 vs. DAF150 groups, most target genes were enriched in metabolic pathways and the biosynthesis of secondary metabolite pathways, followed by the biosynthesis of amino acids. However, among the enrichment results of the DAF135 vs. DAF150 comparison, most of the targets were involved in the biosynthesis of secondary metabolites, plant–pathogen interactions, and carbon metabolism pathways. The expression patterns of four target genes, including *MYB123*, *SCL6*, *AGO1*, and *AGO1B*, were inversely correlated with their miRNAs. However, the expression level of miR156x gradually increased during seed development, whereas the expression level of *SPL14* decreased at DAF135, and reached its highest expression level at DAF150. 

## 4. Discussion

Pecan is a valuable oil-bearing species with high nutritional value. Increasing the oil yield is a key priority for the development of pecan breeding [15]. We found that the oil content was significantly increased during the pecan embryo development (Figure 1B), and a similar phenomenon was found in other oil-bearing nuts, such as walnut and Chinese hickory [14,26]. In Chinese hickory, there was a rapid increase from 95 to 108 days after pollination and a rapid increase in oil content from DAF120 to DAF135 in all three pecan cultivars [26]. Interestingly, the concurrent decline in the soluble sugar content from DAF120 to DAF135 in ‘Pawnee’ suggested a potential relationship between sugar utilization and oil accumulation in pecan seeds (Figure S1). An FA compositions analysis revealed that oleic acid was the predominant FA component in pecan embryos throughout all five developmental stages (Figure 1C), with notable implications for fatty acid biosynthesis. Several key genes, including *ACCase*, *KASII*, *DGAT1*, and *LACS4*, might be involved in the oleic acid content [5]. Moreover, oleic acid (c18:1) is a crucial intermediate of fatty acid biosynthesis and is produced from stearic acid (18:0) [27].

MicroRNAs are a class of single-stranded small non-coding RNA; previous studies established that miRNAs regulate the expression levels of their targets via mRNA degradation or translation inhibition [28]. MicroRNAs have been studied for several years; however, the roles of miRNAs in the process of seed development have been poorly investigated. High-throughput sequencing technology is an experimental approach, which is a rapid, efficient, and convenient way to sequence small RNAs. Most of the previous pecan miRNA studies focused on the graft healing process in pecan, while their specific roles in pecan seed development, particularly in fatty acid synthesis, have been underexplored [16,17]. To gain further insight into the potential roles of miRNAs in the seed development and oil accumulation processes in pecan, a high-throughput sequencing method was employed to compare the differentially expressed miRNAs associated with lipid biosynthesis in pecan seed samples at different developmental stages. In this study, nine small RNA libraries at three stages were constructed, 365 known and 321 novel miRNAs were identified through a bioinformatics analysis, and these known miRNAs were classified into 296 families (Table S3). A total of 378 conserved known microRNAs were found in the pecan genome [7]; however, only 47 conserved miRNAs and 39 novel miRNAs were previously detected in the graft healing process of pecan, highlighting the specificity of miRNA expression in different physiological processes [16]. In total, 204 known and 554 novel miRNAs were predicted during walnut oil accumulation [14]. For another oilseed plant, sesame, 354 known and 671 novel mature miRNAs were obtained from 12 libraries via small RNA sequencing [29]. A length distribution analysis of the miRNA sequences revealed predominant lengths between 21 and 24 nt (Figure 2), which was consistent with our previous results [17]. Similar results were also reported for linseed flax and walnut [14,30]. This length preference aligns with the typical size range of functional miRNAs, underscoring the conservation of miRNA characteristics across different plant species.

The expression patterns of microRNAs can help elucidate the roles of microRNAs in various biological processes [31]. We further analyzed the expression patterns of miRNAs during pecan seed development, and the differential expression patterns observed across various developmental stages suggested a dynamic regulatory landscape (Figure S3). Surprisingly, most known miRNAs exhibited low expression levels, whereas novel miRNAs performed relatively high expression levels; however, opposite expression trends were found in the graft healing process of pecan [16]. In total, 91 known and 181 novel miRNAs were differentially expressed during the three seed development stages (Figure 4), whereas 16 known and 13 novel miRNAs were differentially expressed during the graft healing process [16]. These identified DEMs might play important roles during pecan seed development and oil accumulation. We further analyzed the expression trends of these DEMs, which were classified into eight groups with various expression patterns (Figure 5). Interestingly, more DEMs were up-regulated than down-regulated during the oil accumulation process, which was consistent with previous results in other oilseed plants such as walnut and sesame [14,29]. 

Degradome sequencing has emerged as an effective way to investigate the targets of miRNAs, and a deeper analysis of the identified miRNA targets has shed light on their functional significance [27]. For example, the down-regulation of key transcription factors and enzymes involved in oil biosynthesis indicated a direct regulatory role of specific miRNAs in controlling the oil accumulation in pecan seeds. According to the qRT–PCR and degradome sequencing results, we identified five miRNA–mRNA modules (miR858-y-MYB123, miR156-x-SPL14, miR171-y-SCL6, miR168-x-AGO1, and miR168-x-AGO1B) with potential functions in seed development and oil biosynthesis (Figure 7). Transcription factors play central roles in plant growth, development, and various physiological processes by binding to the promoter regions of their targets to activate or suppress gene expression [32]. Plant oil biosynthesis is physiologically connected to the seed development process, and both processes are affected by key transcription factors, such as WRI1, LEC1, ABI3, and FUS3 [33]. In tea oil camellia, miR858 is involved in regulating seed development by targeting MYB transcription factors, including *MYB3*, *MYB44*, and *MYB82* [34]. MYB genes have been shown to function in the biosynthesis of seed oil, and in *Vernicia fordii*, *VfMYB* played a key role in tung oil biosynthesis. The overexpression of *VfMYB36* in *Arabidopsis* led to a notable increase in the seed oil content [35,36]. The miR156 family is highly conserved, and it is also the most abundant miRNA in plants; miR156 can target SQUAMOSA PROMOTER-BINDING PROTEIN-LIKE (SPL) members to play roles in various biological processes [37]. miR156 participated in the early morphogenesis of embryos via regulating *SPL10* and *SPL11* in *Arabidopsis* [38]. Bna-miR156 regulated early embryo development by targeting SPL, and it affected the seed oil content of *Brassica napus* [39]. Lpu-miR171 and its target SCARECROW-LIKE 6 have been proven to play important regulatory roles in lily somatic embryogenesis [40]. In tea oil camellia seeds, miR171 was involved in seed development by targeting the SCL16 gene [34]. An integrated analysis of miRNAs and mRNAs demonstrated that miR168 affects seed size and lipid biosynthesis in sea buckthorn seed [41]. miR168, which binds to *Argonaute1* (AGO1) and performs post-transcriptional gene silencing, leads to pleiotropic developmental in *AGO1* mutation plants, since this gene regulates the seed maturation process [42]. Except for these four microRNAs, several other miRNAs are also involved in seed development and oil biosynthesis processes, and we have analyzed their expression via qRT–PCR experiments (Figure 6). For example, miR167A has been shown to target the auxin response factor (CsARF8) to affect the fatty acid metabolism in camelina (*Camelina sativa*) seeds, and the overexpression of miR167 changed the fatty acid composition and improved the seed size [43]. 

This study elucidated the regulatory modules associated with miRNAs and their targets, which were related to oil biosynthesis and accumulation in pecan seeds. On the basis of the bioinformatic and expression analyses, we hypothesized that these five regulatory modules might play pivotal roles in seed development and oil biosynthesis in pecan seeds. However, the biological functions of key miRNAs and their targets in pecan should be further investigated.

## 5. Conclusions

In conclusion, we presented the genome-wide identification of pecan seed development and oil biosynthesis through the application of small RNA sequencing. Oil biosynthesis in plant seeds is a complex biological process, and the expression patterns of the identified miRNAs and target genes will help to identify the central regulatory modules involved in this process in pecan seeds, which provides a foundation for future studies. Our findings enhance the understanding of pecan biology and offer potential targets for genetic manipulation to improve the agronomic traits in pecan seeds.

## Figures and Tables

**Table 1:** Distribution of miRNAs in nine libraries.

Samples	Known miRNAs	Novel miRNAs	Total
DAF120	152	317	469
DAF135	189	320	509
DAF150	185	314	499
Total	365	321	686

## Data Availability

The raw sequencing data were submitted to the NCBI with the BioProject including PRJNA1144794 (transcriptome sequences), PRJNA1144852 (small RNA sequences), and PRJNA1152143 (degradome sequences).

## Supplementary Materials

The following supporting information can be downloaded at: https://www.mdpi.com/article/10.3390/foods13182934/s1. Figure S1: soluble sugar content in the development embryos of pecan; Figure S2: Pearson correlation between different samples; Figure S3: expression patterns of known (A) and novel (B) miRNAs during seed development in pecan; Figure S4: analysis of miRNA first nucleotide bias; Figure S5: expression patterns of novel DEMs during pecan seed development; Figure S6: KEGG enrichment analysis of target genes in three seed development stages of pecan; Table S1: specific primers for qRT–PCR assay; Table S2: clean reads of nine small RNA libraries; Table S3: known and novel miRNAs identified during pecan seed development; Table S4: basic information of novel miRNAs; Table S5: differentially expressed miRNAs; and Table S6: predicted target genes of the identified miRNAs.
