# Supplementary material for: The Integrated Analysis of miRNome and Degradome Sequencing Reveals the Regulatory Mechanisms of Seed Development and Oil Biosynthesis in Pecan (Carya illinoinensis)
Source: Foods. 2024 Sep 16;13(18):2934. doi: 10.3390/foods13182934 (PMC11430883; doi:10.3390/foods13182934)
Supplement: Supplementary file 1 [file foods-13-02934-s001.zip › foods-3155941-supplementary/Supplementary Figures.pdf]

Figure S1

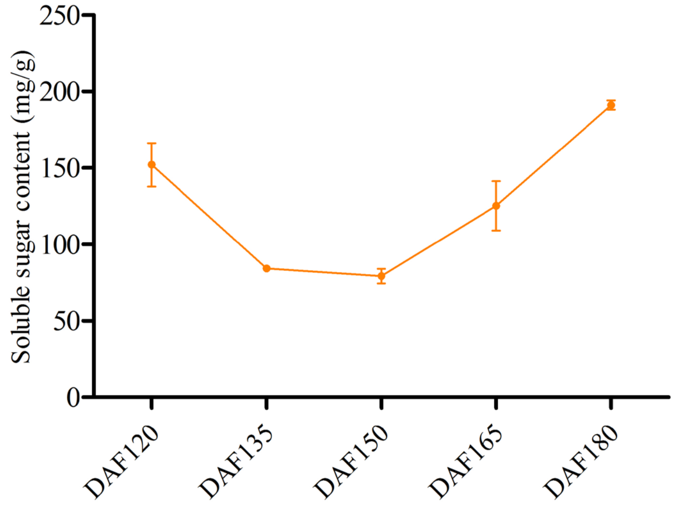

Figure S1 | Soluble sugar content in the development embryos of pecan.

Figure S2

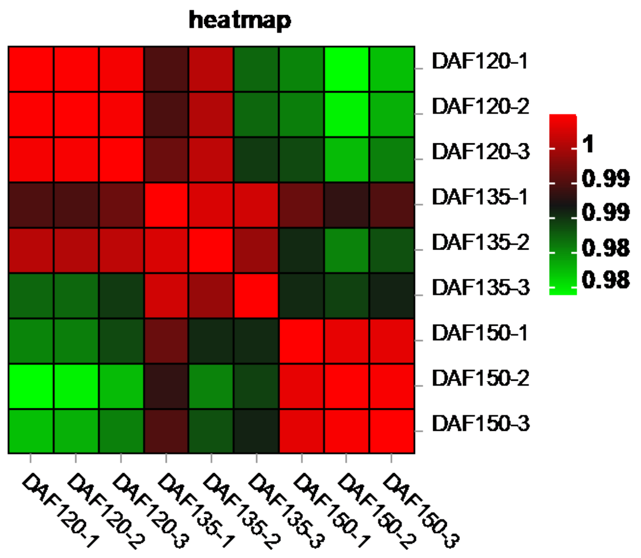

Figure S2 | Pearson correlation between different samples.

Figure S3

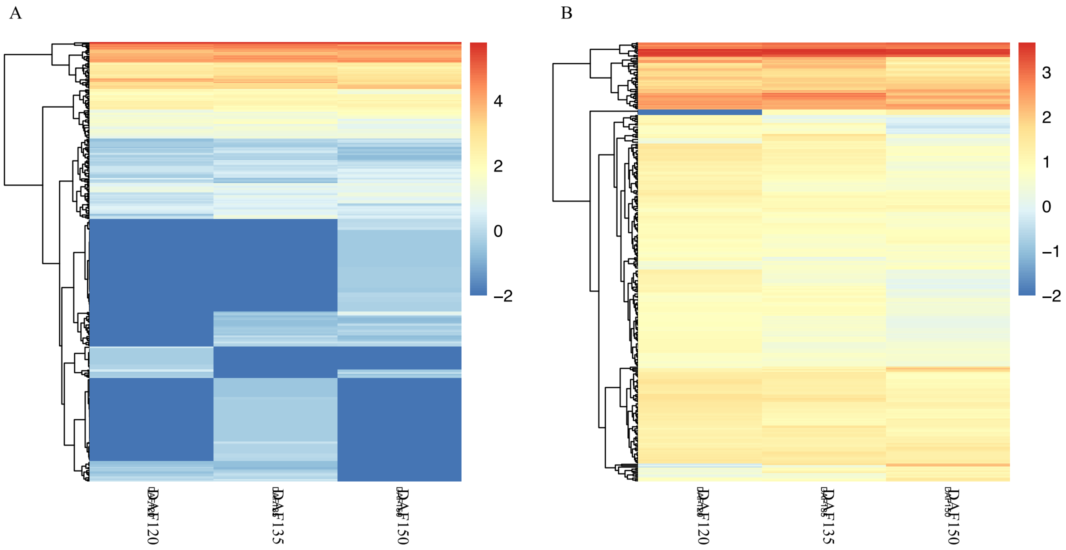

Figure S3 | Expression patterns of known (A) and novel (B) miRNAs during seed development in pecan.

Figure S4

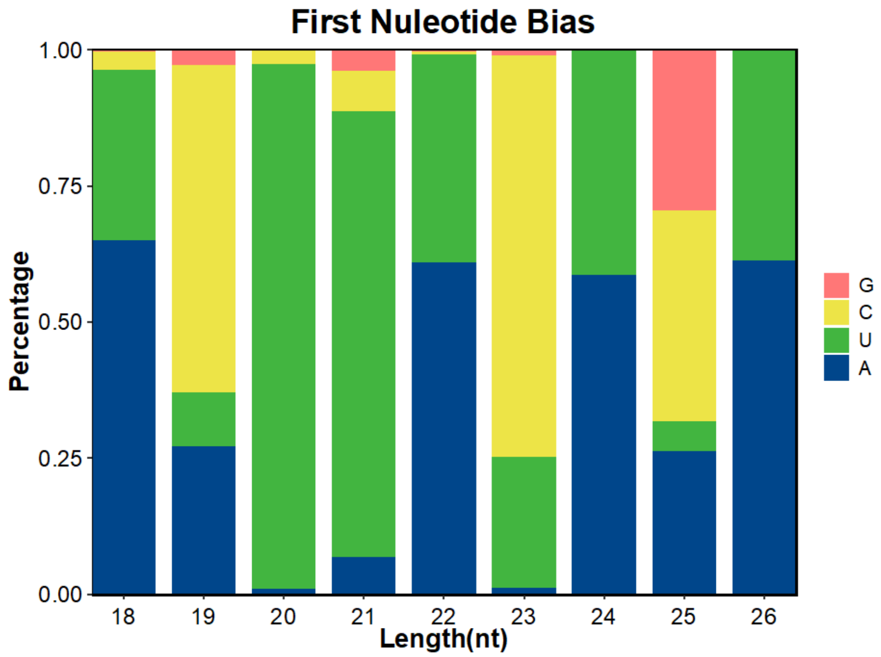

Figure S4 | Analysis of miRNA first nucleotide bias.

Figure S5

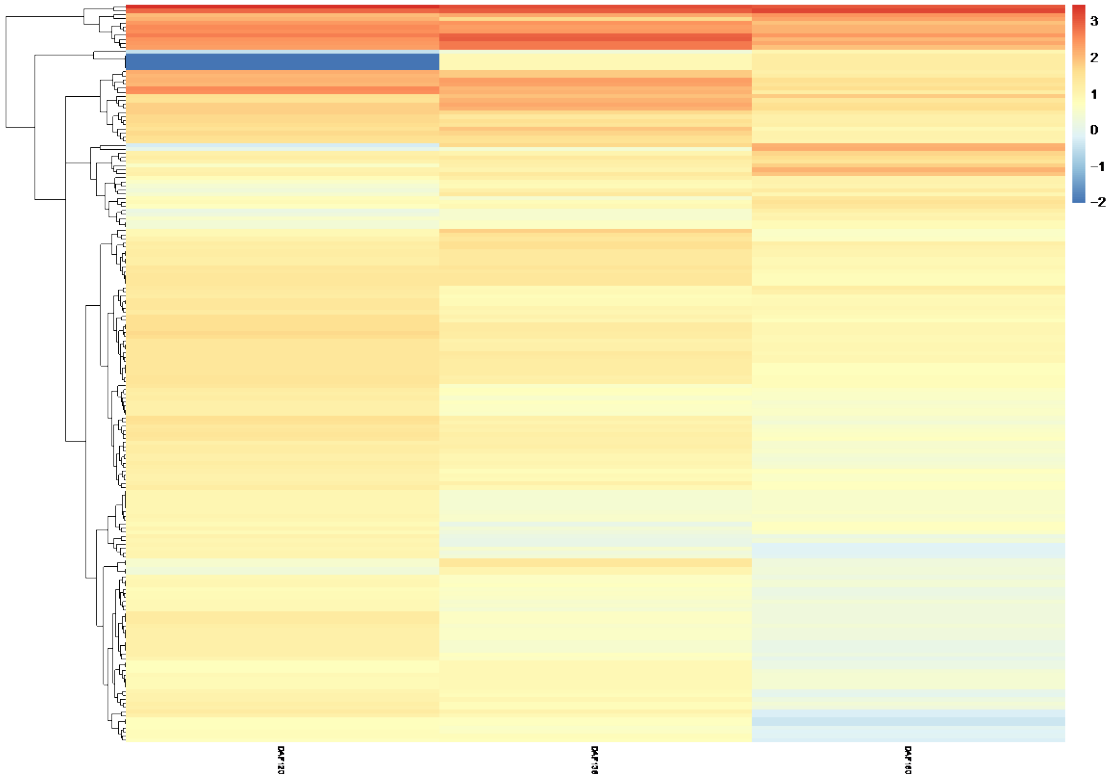

Figure S5 | Expression patterns of novel DEMs during pecan seed development.

Figure S6

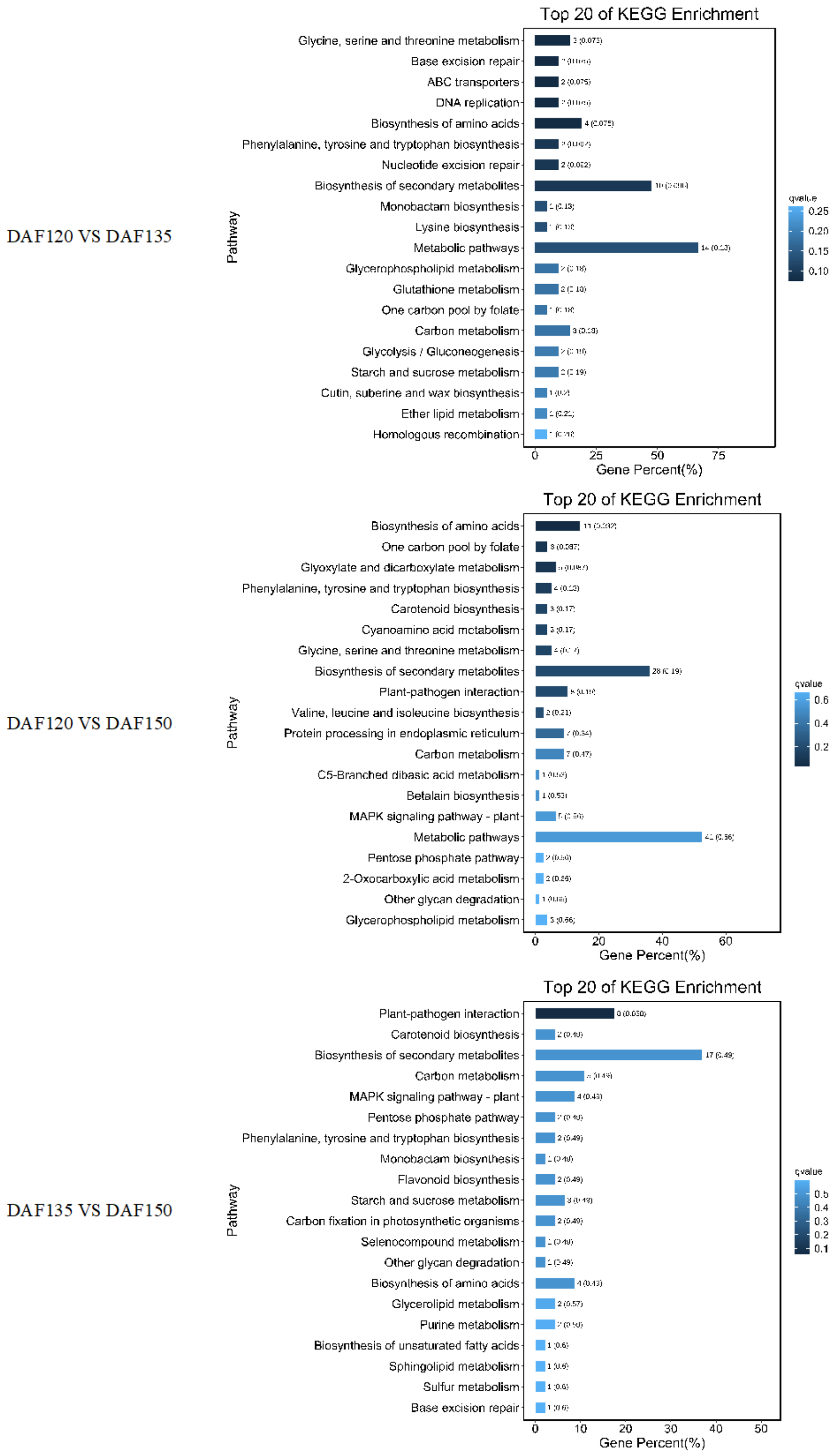

Figure S6 | KEGG enrichment analysis of target genes in three seed development stages of pecan.
